# Multiple rainfall event pollution transport by sustainable drainage systems: the fate of fine sediment pollution

**DOI:** 10.1007/s13762-016-1177-y

**Published:** 2016-11-23

**Authors:** D. Allen, S. Arthur, H. Haynes, V. Olive

**Affiliations:** 1 Heriot-Watt University, Edinburgh, Scotland, UK; 2 Scottish Universities Environmental Research Centre, Glasgow, Scotland, UK

**Keywords:** Sustainable urban drainage systems, Sediment transport, Rare earth tracer, Pollutant treatment efficiency, Stormwater quality

## Abstract

A key design criterion of sustainable urban drainage systems is to mitigate urban stormwater pollution. Current research defines sustainable urban drainage systems (SuDS) pollutant treatment efficiency through the detention of total suspended solids, urban nutrients and heavy metal pollutants within the system during a design flow event, with research focusing on sand (>2 mm) sediment movement. The impact of multiple rainfall–runoff events on the fine sediment (<2 mm) treatment efficiency of SuDS is not yet well defined, and the temporal movement of detained sediment has not been investigated in detail. The field research presented in this paper addresses this research gap, monitoring ongoing fine sediment transport through a best-practice-designed SuDS network over 12 months through the use of a novel rare earth oxide trace methodology. Through time-stepped monitoring of the fine sediment pollution across three SuDS treatment trains (networks), the following key conclusions have been drawn. (1) That fine sediment becomes re-suspended and re-deposited within SuDS assets and the network as a result of ongoing multiple rainfall–runoff events. (2) That this re-suspension continues for over 52 weeks. (3) That by area, linear wetlands (within the monitored networks) outperform wetland and swale assets in multiple event fine sediment detention. And (4) that multiple event monitoring and analysis of fine sediment within a SuDS network highlights the under-performance of SuDS assets against current design event expectations.

## Introduction

Sustainable urban drainage systems (SuDS) have been implemented within urban development environs to convey and treat urban stormwater (Woods-Ballard et al. 2007). Urban development creates impervious spaces that prevent infiltration of stormwater runoff into the soil, thereby increasing the runoff into downstream watercourses. The use of land for urban purposes, residential living, commercial development and industrial business, creates a concentration of heavy metal and sediment pollutants that are collected from urban impervious surfaces and conveyed into neighbouring watercourses by the stormwater flow (Sekabira et al. 2010).

Understanding long-term sediment conveyance–detention processes in sustainable urban drainage systems (SuDS) is key to quantifying the contaminant risk and potential flood storage loss within the urban environment drainage network. Recent studies have assessed both event-based suspended solid mitigation by SuDS assets and annual sedimentation budgets within wet assets (Wong et al. 2006; Deletic 2004). However, no data exists that explains the variability of conveyance–detention over multiple, consecutive events. Similarly, the long-term functionality of ephemeral SuDS assets or blue–green treatment trains is not well understood.

The majority (>85%) of urban contaminants, pollutants including heavy metals and nutrients, are adsorbed to sediment and thus conveyed through the urban stormwater network as sediment is moved (Jones et al. 2008; Saeedi et al. 2004). Urban pollutants such as copper, manganese, nickel and zinc adsorb easily to suspended and deposited sediment of 250 µm and smaller in size (Saeedi et al. 2004). Thus, monitoring fine sediment transportation through the SuDS network provides an effective indication of both sediment detention and pollutant (such as heavy metals) detainment within the vegetated sustainable drainage system. Research undertaken by Deletic and Fletcher (2006) illustrated that vegetated grass filter strip treatment achieves a performance of 60–85% total suspended solid (TSS) removal during a single runoff event. Hossain et al. (2005) field analysis reports detention pond TSS removal efficiencies of 68–99%. Birch et al. (2004) presented a wetland removal potential (TSS reduction) of 46–98%. Backstrom (2002) undertook field testing of vegetated swales and found the runoff event TSS removal efficiency to range significantly, but to generally provide 80–90% removal. Each of the aforementioned treatment efficiencies is runoff event specific. Multiple event analysis of SuDS pollutant treatment efficiencies has not yet been studied in detail. However, SuDS are expected to function to a design capacity, for example, sediment volume removal rate for a wetland 55%, pond 80% swale 75%, filter strip 55% (Leisenring et al. 2013) over their life cycle of up to 25 years. The influence of multiple events on sediment pollutant transport may result in multiple event variability of efficiency. The long-term treatment efficiency of SuDS assets and a SuDS treatment train or network generally assumes that each runoff event will achieve the desired treatment efficiencies with no influence of hysteresis from previous runoff events or event consequences.

The research presented in this paper has been designed to address this knowledge gap and further the understanding of sediment pollutant transport through a SuDS network over multiple runoff events. The field research site is located in Bathgate, Scotland and field work occurred during 2014. A novel sediment tracer methodology, the use of rare earth oxide (REO) tag and monitoring of urban fine sediments, has been used to trace sediment from specific urban sources into and through established SuDS networks. Using this novel trace method, sediment from unique release locations and release time periods have been tracked through established SuDS networks over 12 months. The SuDS networks were sampled fortnightly, collecting both surface flow samples and bed deposition (through sediment traps) for each SuDS asset. This has provided a spatial and temporal trace sediment dataset through which multiple rainfall–runoff event sediment resuspension and transport can be defined.

## Materials and methods

**Fig. 1:**
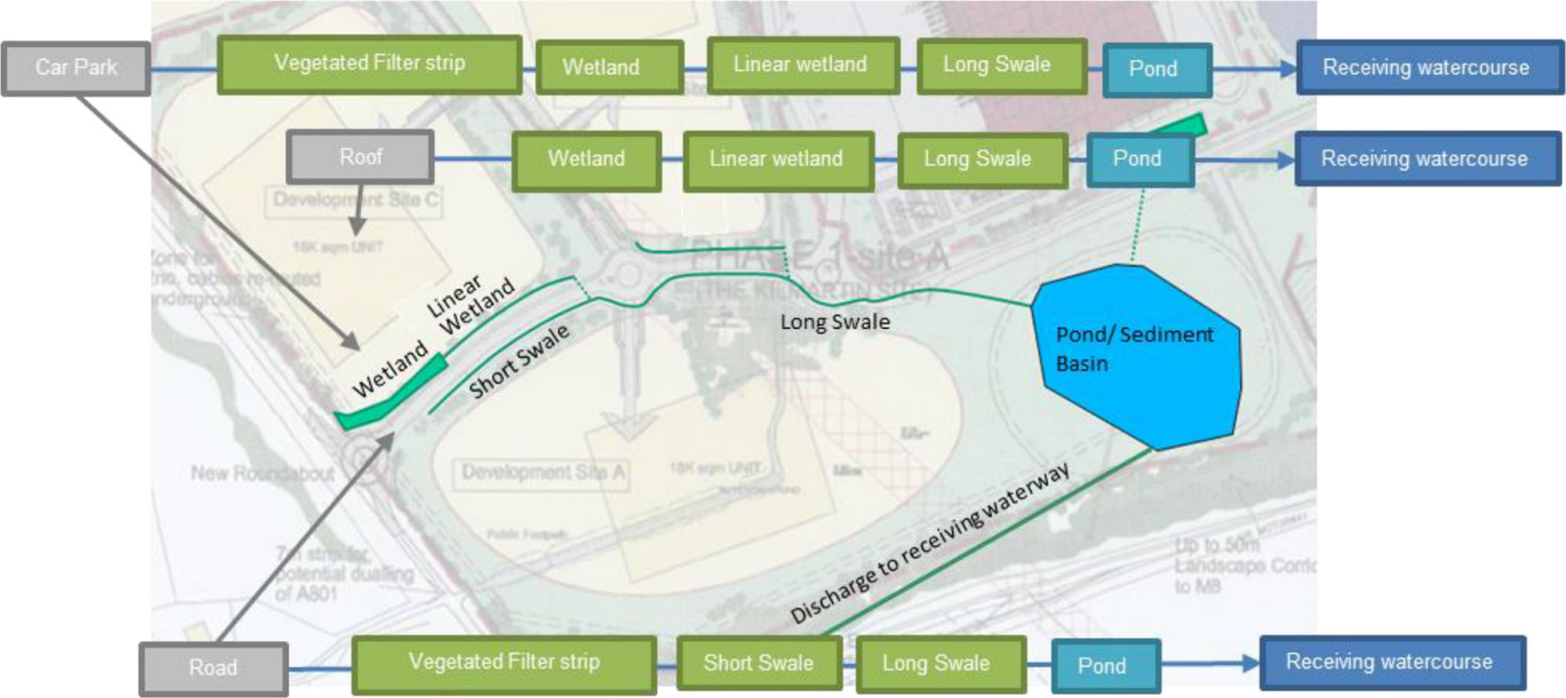
Schematic of J4M8 SuDS networks and key urban pollution surfaces

The J4M8 distribution park (located in Bathgate, Scotland) incorporates a set of established and well-maintained SuDS treatment train networks. This commercial area has been designed as a ‘pipe-less’ development, conveying all stormwater via vegetated surface measures to the legal point of discharge, the River Almond. The SuDS assets within J4M8 comprise of vegetated filter strips (VFS), vegetated swales, linear wetlands, a wetland and a pond. Figure 1 provides an overview of the SuDS networks and the three urban pollutant surfaces considered in this field research.

Suspended concentration and bed-deposited sediment mass were monitored fortnightly over 12 months to provide a fine resolution (temporal and spatial) dataset of multiple runoff event sediment transport. The sampling interval was specifically designed to capture as many sample points as physically and economically viable over a 12-month period. Daily sampling would have provided a more detailed dataset but at the cost of a higher fine sediment and REO trace removal. Monthly sampling was considered too coarse a time step, with a higher likelihood of the REO tagged sediment passing without detention in the traps of surface flow samples. Therefore, given the economic and physical time constraints on sampling, the fortnightly sampling regime was adopted with acknowledgement that a smaller sampling time step may provide more detailed results.

Rainfall, flow depth, flow velocity and tracer sediment monitoring data from car park, roof and road sources was collated to assess the performance of four SuDS assets (wetland, linear wetland, short and long swales). Collated, these data permitted detailed analysis of sediment deposition potential, distribution, residence and flushing efficiency for both individual SuDS assets and the whole system.

Rainfall data were collected as it fell, while flow depth velocity data were collected every 15 min and sediment sampling occurred every 14 days. It is acknowledged that these datasets initially lack synchronicity, requiring modification of both rainfall and flow datasets to support the sediment sampling occurrence. Thus, rainfall, flow depth and velocity were condensed to 2-week total, average and maximums and event occurrence values. A second dataset considering the antecedent dry period, most recent rainfall–runoff, flow depth and velocity at the time of sediment sampling was also created. Considering the average rainfall intensity, flow depths and velocities cause a potential dilution in detail in the dataset, and for the purposes of this field research, this limitation and modified dataset was considered sufficient for trace sediment transport purposes.

Surface runoff samples were collected using an automated sampling system (providing pipe flushing prior to sample acquisition) from each surface sample location within the SuDS network. Samples were collected from within the main flow path. Bed deposition was collected using sediment traps placed below the surface sample locations. Sediment traps were designed, using Van Rijn (1984) saltation assessment, to ensure material up to 2 mm in particle size was collected over the 2-week sampling period. Sediment traps were set into the bed of all SuDS assets, maintaining the level bed surface where sediment traps were located, and were supported by core samples of bed material.

Fine sediment was tagged using unique rare earths. Tagged sediment was released from three specific locations: on a specific area of car park within the distribution centre, within the downpipe from the roof runoff of the distribution centre building, and on the internal road surface (indicated in Fig. 1). Sediment, equivalent to 1/12th of the annual sediment pollutant load for this urban area, was tagged using rare earth element tracers. Three separate sediment volumes were created, for release onto the three separate car park, roof and road locations, each using a unique individual rare earth tracer. The REO tracers used for the car park, roof and road were Nd, Sm, Gd, Tb; Y, La, Ce, Pr; Dy, Ho, Er, Yb, respectively. The sediment was tagged following the detailed methodology described by Zhang et al. (2001, 2003), at a tracer concentration rate of 10 g/kg of sediment (Allen et al. 2015), and released evenly onto the urban surfaces only once at the beginning of the sampling period. Tagged sediment was designed to mimic naturally occurring urban sediment pollution, in both mass and particle size distribution (PSD). Sediment size ranged between 0.45 µm and 2 mm, with a d50 of 60 µm. Tagged sediment, once released, was left to move naturally off the urban surface (roof, car park or road) via rainfall–runoff events, into and through the SuDS network.

## Results and discussion

### Rainfall and flow characteristics for the sample period

The site-specific rainfall was monitored adjacent to the wetland. Three ‘Stingray’ depth and velocity meters provided continuous flow monitoring within the SuDS network, within the wetland, within the linear wetland and within the swale. The field work commenced mid-winter (January).

The fortnightly rainfall ranged from 0 to 98 mm in total, with an average fortnightly rainfall total of 36 mm (SD 30). The number of rainfall events within the fortnightly monitoring periods ranged from 0 to 24, with an average of 10 rainfall events per fortnight (SD 6.5). Antecedent dry days (ADD), the period of no rainfall, within the fortnight ranged from 0 to 13 days, and the average ADD over the fortnight was 8.5 days (SD 3.4). The period of no rain prior to an event sample was 21 h on average (SD 25, range 0–90 h) with this event lasting on average 2 h (SD 4.6, range 0–23 h).

Weeks 38 and 46 show the greatest rainfall over the 2-week period prior to sample collection (90.2 and 85.2 mm, respectively). This coincides with high event occurrence (10–12 individual rainfall events), a dataset correlation of 0.4. The average rainfall intensity over the 2-week period prior to sampling is less varied (0.43–12 mm/hr) than the rainfall intensity of the event directly prior to the sampling (0.7–30 mm/hr). The antecedent dry period within this 2-week sample period also varied considerably (0–14 days). In general there is a large amount of variation in rainfall (event and total overall) across each sample period, therefore potentially creating high variability in sediment suspension and deposition within the SuDS treatment train over this time.

### Suspended and deposited sediment across the SuDS treatment train

**Fig. 2:**
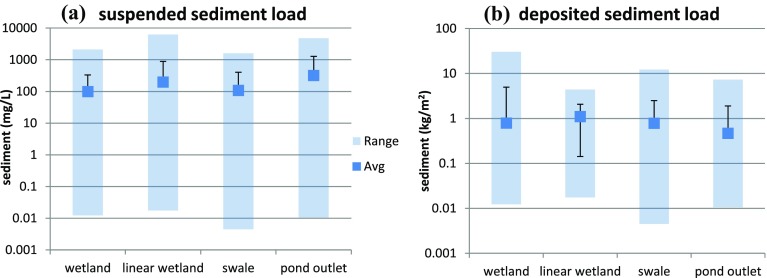
Surface TSS concentrations (**a**) and bed deposition mass (**b**) for the sample period. The range is illustrated by the *blue bars*. Average values (*dark blue box*) and SD are also presented

Alongside the rare earth tracer analysis undertaken through the J4M8 SuDS networks, the total suspended solid (TSS) and deposition mass for each sample location were also monitored. The collected surface stormwater samples were filtered through a 0.45-µm filter, dried and weighed following the BS ISO 5667-6:2014 methodology. Over the monitoring period, the concentration of suspended solids within the SuDS networks (Fig. 2a) was greatest within the linear wetland (196 mg/L) and lowest within the grassed swale (107 mg/L). A general trend was found illustrating the influence of a blue (wet) environment and vegetation. SuDS assets with standing or flowing stormwater showed a generally higher TSS concentration than their ephemeral counterparts. The closer the stormwater surface level proximity to the vegetation height (i.e. where stormwater was at or below the top of SuDS vegetation) the greater the average TSS concentration.

With regard to sediment deposition (Fig. 2b), the largest range of deposition occurred within the wetland (0.01–30 kg/m^2^, average of 0.79 kg/m^2^). This may be because of the direct roof runoff inflow entering the wetland below the standing water level and causing exacerbated resuspension of material during rainfall–runoff events or the location of the wetland at the upstream end of the SuDS network for both the roof and car park runoff. The swale samples illustrated the second greatest variation in deposition (0.001–12 kg/m^2^) but a very similar average deposition rate to that of the wetland (0.78 kg/m^2^). This may result due to the assets location within the SuDS treatment train, but may also be due to the short vegetation and higher conveyance capacity of this type of SuDS asset. The average deposition within the wetland is notably lower than that of the linear wetland (1.1 kg/m^2^). By area (m^2^) the linear wetland is shown to be the most efficient (by up to 41%) in temporary sediment deposition (deposition on the bed of the SuDS asset).

### Sediment transport through the SuDS network

Samples from both the surface flow and the sediment traps (bed deposition) were collected fortnightly throughout the SuDS treatment train. The sampled sediment was prepared for REO trace analysis using strong acid digestion and then tested using an inductively coupled plasma mass spectrometer (ICPMS) to determine the concentration of rare earth tracer in each sample. An ICPMS provides metal concentration analysis at parts per billion concentration, allowing very small concentrations of material to be analysed. Using the individual rare earth tracer signatures applied to separate sediment volumes released from the car park, roof and road area within J4M8, the movement of sediment within the SuDS treatment train was monitored.

Sampling was undertaken across the entire SuDS network, at multiple locations within each of the SuDS assets. Each sample site (both the sediment trap and corresponding surface flow sample point) was located to be representative of a short reach of SuDS asset. The REO tagged sediment found in each surface and sediment trap sample was assumed to be representative of the corresponding reach and using this assumption a sediment balance was created for the SuDS networks.

The mass of REO tagged sediment remaining on the urban surface was sampled fortnightly, in conjunction with surface and bed deposition sample occurrences. There is an assumption made that the area sampled was representative of the total urban surface. However, it is acknowledged that validation of this assumption is not possible without total surface sampling and tagged sediment replacement. Thus, the data presented in Table 1 are the most accurate representation of the urban surface fine sediment release for the field study site over this monitoring period.

**Table 1:** REO tagged sediment balance within the three SuDS networks

Monitoring period	Release	Week 2	Week 8	Week 16	Week 24	Week 32	Week 40	Week 48	Week 52
Roof									
Cumulative mass detained in system (%)		99.9	99.9	99.9	99.8	99.7	99.1	99.1	98.4
Cumulative detention in system (g)		600	3246	4296	4892	4983	4951	4955	4921
Remaining release on urban surface (g)	5000	3400	1750	700	100	0	0	0	0
*Of which*									
Suspended total at time of sampling (g)		273	77	6	0.1	2	27	14	4
Deposition due to resuspension (g)		12	1020	1102	839	889	929	1491	318
Car park									
Cumulative mass detained in system (%)		99.9	99.9	99.9	99.7	99.7	99.2	97.5	97.0
Cumulative detention in system (g)		2937	11,543	17,831	20,305	19,886	20,481	20,372	18,180
Remaining release on urban surface (g)	21,000	18,606	9450	3150	2100	1050	315	0	0
*Of which*									
Suspended total at time of sampling (g)		106	159	0.04	0.2	0.3	189	88	72
Deposition due to resuspension (g)		0	0	0	1301	91	481	344	105
Road									
Cumulative mass detained in system (%)		96.5	89.7	91.9	91.6	89.6	88.1	84.6	83.5
Cumulative detention in system (g)		868	3229	5055	5441	5376	5283	5074	5012
Remaining release on urban surface (g)	6000	4200	1560	300	7	0	0	0	0
*Of which*									
Suspended total at time of sampling (g)		450	179	8	7	13	48	17	16
Deposition due to resuspension (g)		66	2389	477	691	688	499	824	124

The values are presented as grams and percentages of tagged sediment within the SuDS network

Average material lost to sample activities is 0.34 kg/year, SD 0.31

The REO tagged sediment did not totally wash off the urban surfaces and enter the SuDS network with the first rainfall–runoff event. This wash-off rate is dependent on rainfall event frequency, rainfall intensity and surface design (slope, roughness) and is therefore variable according to site characteristics. Table 1 illustrates that the majority of material (over 70%) was conveyed from the urban surfaces by stormwater into the SuDS network within the first 10 weeks.

REO tagged sediment was washed off the three urban surfaces at differing rates, with the road wash off occurring at the fastest rate. The majority (90%) of tagged roof sediment was removed from the roof surface over the first 12 weeks (the final 10% conveyed in following weeks 4–6). This was the fastest urban sediment release. Tagged sediment placed on the roof took five times longer to move into the SuDS network, with 90% of the material released from the road surface within the first 20 weeks. The car park surface was the slowest urban release surface, taking 36 weeks to wash off the surface into the SuDS network, a full 14 weeks longer than the road surface. The extended wash-off time for both road and car park surface can be explained through flow path differentiation. Both the car park and road flow paths are overland, therefore requiring a greater sheet flow, comparative to the roof water piped flow, to entrain and transport this sediment material off these urban surfaces.

Furthermore, the extended release time of car park-sourced material, comparative to road-sourced sediment, may be due to the difference in traffic loading. The annual average daily flow (AADF) of vehicles in west Lothian roads (A801), provided by the Department of Transport (2012), is 12,340 vehicle movements. This is significantly higher than the vehicle movements expected in a commercial car park (approximately 690, a maximum of 4 movements per car space in the field site car park). While the AADF is only indicative for this location, it shows that there is at minimum an order of magnitude of difference in traffic loading. It should also be noted that traffic speeds along the road will reach up to 30 miles/hr, whereas the car park will be closer to 2–5 miles/hr. The elevated vehicle loading and vehicle speed on the road result in a greater pressure on the road surface (type impact) causing road surface particles to disperse as stormwater is forced across the road surface (Oke and Ajayi, 2007).

The first flush through of suspended tagged material occurs generally over the first 8 weeks for material released from roof, car park and road sources. There is a notable rise in suspended REO tagged sediment around weeks 40–48 within all SuDS networks. The network suspended sediment responses are temporally similar. This suggests that there is an influence beyond the SuDS asset and network design that is influencing the ongoing movement of sediment. The occurrence of suspended sediment concentration rise is possibly due to rainfall, flow or temporal elements.

The tagged sediment detention within the SuDS networks is seen to fluctuate over the 52-week monitoring period. None of the monitored systems show a peak deposition occurrence within the first 2 weeks of sampling. Instead, the peak deposition in the sediment traps occurs in the week after the cessation of surface sediment release. Therefore, the surface sediment release is shown to be a key and logical factor in estimation of SuDS asset bed deposition. If an urban surface continues to release fine pollutant sediment into a SuDS network, the deposition within the system will vary, without peaking, until the urban surface is ‘clean’ of sediment or the capacity of deposition has reached its plateau. The detention capacity plateau is a temporal consideration, a method to try and define an average long-term sediment deposition rate for an asset or network. Within this case study, the deposition rates in Fig. 2b could be considered as the deposition plateau within the established SuDS assets. Further research into deposition plateau potential in established ephemeral SuDS assets is required to provide detailed understanding of this process.

The deposition due to resuspension has been calculated by considering the available mass flowing into the sampling location (from the urban surface release, potential upstream bed deposition and suspended sediment), the mass leaving the sampling area and moving downstream and the mass detained in the sediment trap and in suspension at the time of sampling. The first large mass resuspension does not appear to occur in correlation with the urban tagged sediment release of peak suspended sediment occurrences. Thus, the first notable resuspension activity within the SuDS assets may be influenced by more than rainfall–runoff event occurrence and material availability. However, the second notable resuspension activity occurs during week 48, concurrently with the second peak in suspended sediment values. Thus, this second resuspension can be considered to have caused the increased suspended sediment values and be a result of a temporal occurrence (rainfall–runoff occurrence).

The cumulative deposition within the SuDS network fluctuates over the 52-week sample period. Temporary detention within the SuDS network is not stable, and peak detention does not occur at either week 2 or week 52. Roof and road-sourced sediment detention falls slightly but continuously after peaking during week 32 and week 24, respectively. car park-sourced sediment detention within the SuDS system continues to rise until week 40, where the slight but continuous decrease in detention commences. This suggests that while event-specific analysis can provide event-specific water quality treatment or mitigation measures, to understand the actual detention potential of a SuDS network, the system should be monitored for significantly longer (+40 weeks in this location). Furthermore, the slight but continuous decrease in detention during the latter weeks of this monitoring period suggests that the peak detention efficiency seen in a SuDS network is not the long-term detention efficiency.

**Fig. 3:**
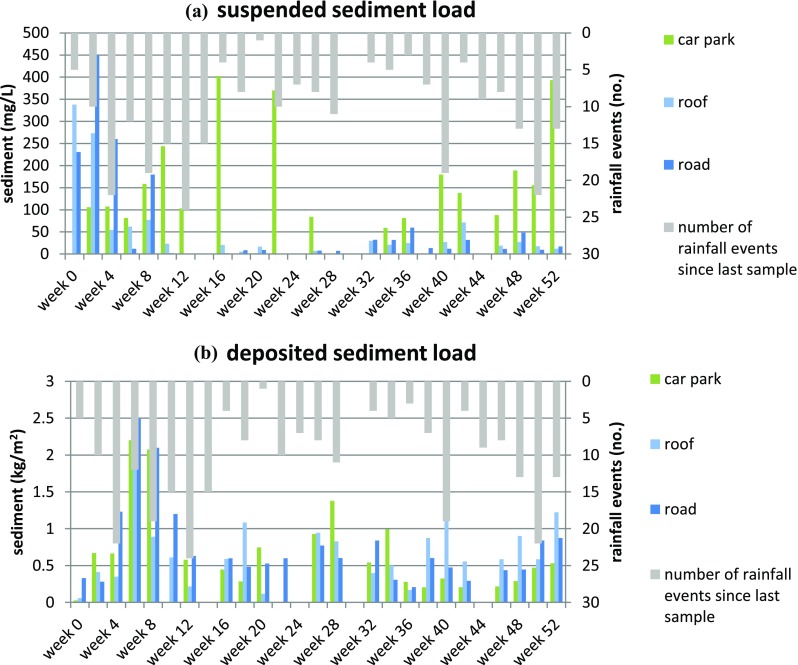
New deposition at each sampling location for surface (**a**) and bed deposition (**b**) rare earth tagged sediment relative to the release location within J4M8

**Fig. 4:**
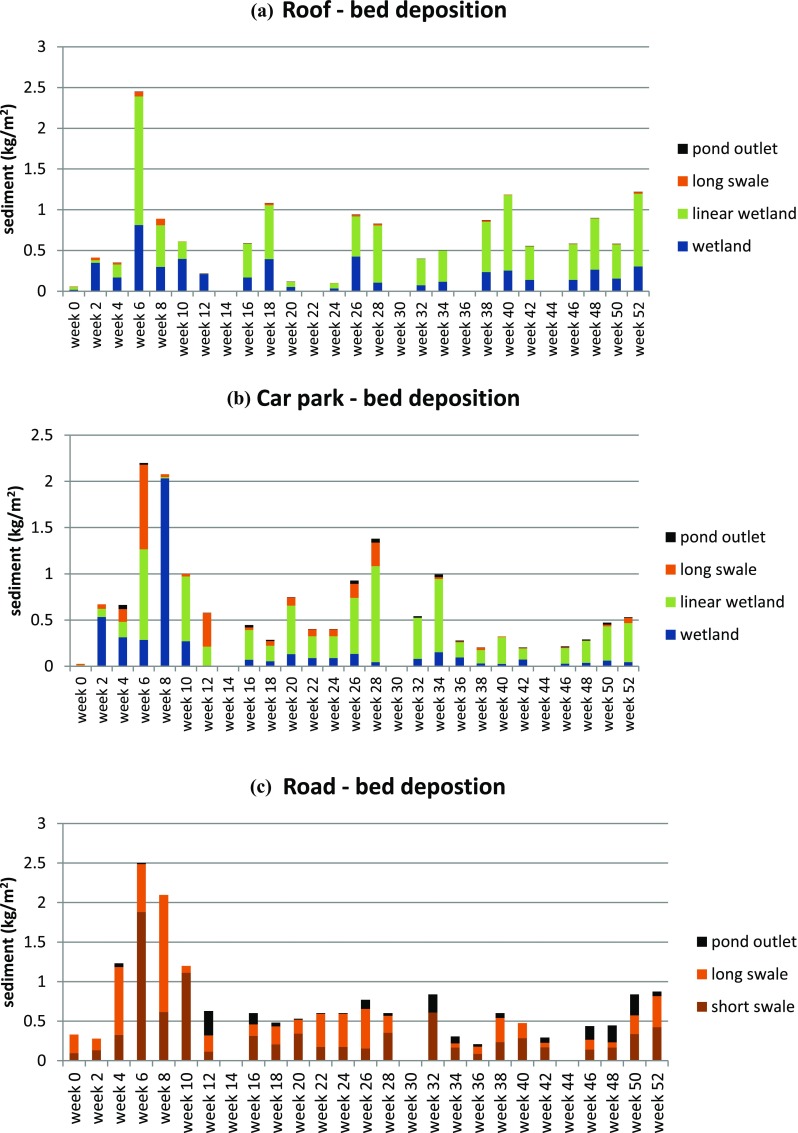
Spatial and temporal deposition of tagged sediment

The graphs in Fig. 3 show the sediment trace concentrations within the SuDS treatment train from the three key urban sources relative to the rainfall events. Figure 4a provides a summary of the number of rainfall events occurring during the preceding fortnight. Using trace concentration monitoring through this network, the movement of sediment through the SuDS treatment train has become visible.

Sediment is shown to be in suspension (Fig. 3a) within the SuDS network right across the 52 weeks monitored. The car park-sourced tagged sediment has a generally higher concentration in suspension compared to both road and roof runoff after the first 8 weeks. The roof-sourced sediment shows elevated suspended concentrations within the SuDS network during the first 2 weeks, while the road-sourced sediment is found at concentrations over 150 mg/L up until week 10.

The ongoing inconsistent car park-sourced sediment concentrations across the monitoring period may be due to the inclusion of a vegetated filter strip (VFS) in this network. The VFS bordering the car park surface has consistent vegetation planting and an effective design (compared to the VFS along the road). All stormwater runoff and sediment is conveyed over this well-maintained vegetated filter strip prior to entering the wetland. The filter strip temporarily detains and releases fine sediment from the car park surface into the wetland, thus potentially causing the ongoing elevated concentration levels in the car park surface sample dataset.

The roof-sourced sediment SuDS network incorporates no vegetated filter strip, and sediment-laden stormwater is discharged directly into the wetland (sub-surface pipe discharge). As a result, there is limited extension in significantly elevated roof-sourced sediment concentrations in suspension (>100 mg/L). The road-sourced sediment SuDS network does include a VFS, but it is poorly maintained and has a low vegetation density. It is suggested, on review of the results in Fig. 3a, that this filter strip provides some temporary detention of road-sourced sediment, but to a lower level provided by the car park VFS.

The sediment shown in suspension (through sampling of the SuDS networks flow) is mirrored to some extent in the deposited sediment load. It should be noted that there is a lag between elevated suspension concentrations and elevated bed deposition of 4–6 weeks. The suspended sediment appears to react directly to individual rainfall events, while the bed deposition increases with increasing rainfall occurrence (a greater number of rainfall events).

Of key interest in Fig. 3a, b is that there is still notable suspended tagged sediment and tagged sediment deposition within the SuDS networks across the entire 52-week monitoring period. 99% of the tagged sediment is conveyed off the urban surfaces after 24 weeks. Thus, the tagged sediment material shown across the second 6 months is the result of ongoing resuspension and deposition of tagged sediment within the network.

### Asset-specific sediment deposition within the SuDS network

The spatial deposition of tagged sediment is illustrated in Fig. 4(a–c). Tagged sediment is shown not only to pass through the SuDS networks in suspension (Fig. 3) but also to become deposited downstream of the treatment train (deposition at the pond outlet). Thus, the monitored SuDS networks therefore fail to fully protect the downstream watercourse from the urban land use influence (polluted stormwater), allowing up to 17% of the tagged sediment to be suspended or become deposited at the downstream outlet of the pond (varying according to SuDS network composition and runoff/flow characteristics over the monitoring period).

Road material appears to traverse the length of the SuDS system prior to the pond and primarily become deposited in the swale sediment trap just upstream from the pond (within the downstream end of the long swale). This may be due to the downstream boundary condition of this reach of swale resulting from the standing water presence of the pond, and therefore a slower flow velocity through this section of swale. The decrease in flow velocity supports higher settling rates and the potential for finer particle matter to deposit. When the total short- and long-swale SuDS assets are considered, both temporarily detain moderate quantities of tagged sediment. The short swale detains on average 45% (SD 20%) of the material tagged sediment detained in the road SuDS network, while the long swale detains on average 39% (SD 20%). The increased swale length does not appear to notably benefit the detention efficiency of the swale SuDS asset. This may be due to the more dense vegetation within the short swale and the smaller bed width and stormwater conveyance through the asset.

car park-sourced tagged material is shown to deposit throughout the SuDS network. Moderate relative deposition occurs in the wetland (average 24%, SD 21%). The greatest deposition occurs within the linear wetland (average 57%, SD 24%). Both of these SuDS assets have a lower flow velocity, due to dense vegetation, boundary constraints and flow management design.

The roof-sourced tagged sediment settled predominantly in the wetland (average 37%, SD 21%) and linear wetland (average 56%, SD 24%) (Fig. 4c). A small amount of total tagged material is deposited in the downstream swale extent within the long swale (average 2.7%, SD 2.8 %). There is limited detention within this grassed swale, predominantly due to supply. As with car park sediment, the linear wetland is shown to achieve a greater temporary detention efficiency than the wetland or long swale. The average detention efficiency occurring within the wetland is greater for roof sediment than car park sediment. Roof runoff enters the wetland sub-surface rather than as overland flow. The field results suggest that sediment-laden stormwater may be more effectively treated (a benefit of +20% within the case study wetland) when stormwater enters the wetland sub-surface. Further field tests and more detailed analysis are required to confirm this finding.

**Fig. 5:**
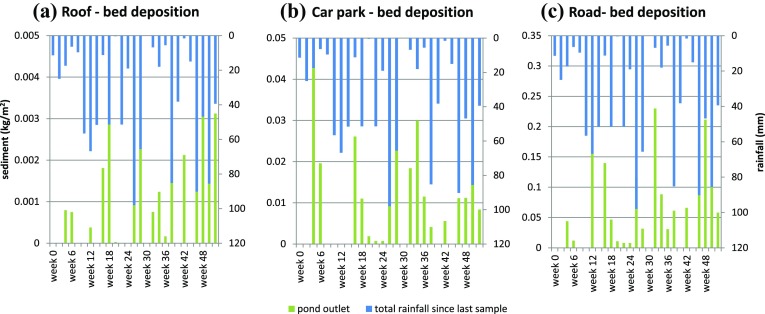
Sediment deposition downstream from the SuDS network, relative to total fortnightly rainfall depth

Roof, car park and road-sourced sediment was transported and deposited at the pond outlet after 4 weeks (Fig. 4, expanded in Fig. 5). Thus, for these SuDS networks it can be seen that sediment traverses the entire length of the system when multiple rainfall–runoff events are considered. While negligible sediment pollution may be seen to reach the discharge point of a SuDS network during a design or single rainfall–runoff event, when considered in the context of a hydrologic series, sediment pollution is carried through and out of the SuDS network. Furthermore, the sediment deposited at the pond outlet does not follow a consistent temporal pattern for all sediment sources (and therefore SuDS networks). Sediment that moves through the short and long swales only (road-sourced material) showed a skew towards later deposition (during weeks 24–52). Material sourced from the car park and roof, passing through the wetland, linear wetland and long swale, showed no specific skew or temporal trend in outlet deposition. Therefore, it may be inferred that the inclusion of the wetland and linear wetland in the SuDS network resulted in a more continuous movement of sediment. This may be due to the more ephemeral nature of the swales relative to both wetland and linear wetland, allowing a quantity of fine sediment material to remain in suspension in the wet SuDS assets thus making this material more easily available for transport during rainfall–runoff events.

The deposition at the pond outlet, the downstream extent of the SuDS networks, does not correlate with the internal network temporal deposition pattern. The car park-sourced sediment deposition at the pond outlet has a generally low correlation with rainfall–runoff event characteristics: rainfall depth prior to sampling (0.3), total rainfall since last sample (0.1), total number of rainfall events since sediment release (0.1), number of rainfall events since last sample (0.04). However, both road and roof sediment deposition at the pond outlet showed moderate correlation with total rainfall since last sample and the total number of rainfall events since sediment release (0.4 and 0.6, respectively). Thus, there is a transport process within the road and roof SuDS networks that is functioning in a notably different manner than the car park SuDS network. The key differences between the car park SuDS network and the roof and road networks are (1) the rate of sediment release from the urban surface and (2) the inclusion of a well-designed and maintained VFS. Thus, both the urban surface release and transport process within a maintained VFS are considered key elements in the determination of fine sediment transport.

### SuDS network influence on detained sediment particle size

**Fig. 6:**
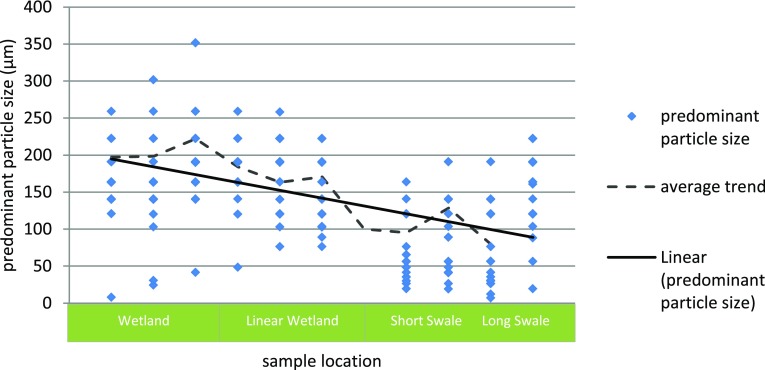
Peak particle size of samples taken from the sediment traps

The particle size distribution (PSD) of bed deposition samples shows a consistent decreasing trend in the size of material deposited within the sediment traps, moving downstream through the SuDS network. Sediment deposited in the wetland is the largest in size (d50 = 197–221 µm). PSD varies across the wetland, but is greater in size than the material deposited within the linear wetland (d50 = 163–183 µm) and grassed swale (d50 = 79–145 µm). The sample particle size structure is mono-disperse, with each SuDS asset within this treatment train collecting deposition of a consistent primary particle size range (illustrated in Fig. 6).

As expected, the larger sediment deposits within the early stages of the SuDS network (within the wetland). Through inclusion of the second and third SuDS assets, the linear wetland and long swale, the finer particles of sediment pollution within stormwater runoff are detained. This result supports the use of SuDS as a treatment train or network rather than individual disconnected assets, allowing sediment pollution of a greater range of size to be detained. In the management of heavy metal stormwater pollutants, those found adsorbed to fine sediment (<250 µm), and detention of this fine sediment is highly important (Jones et al. 2008, Adiyiah et al. 2014). Through implementation of a 3+ asset SuDS system, the finer (d50 < 100 µm) sediment pollutants start to become detained and potentially captured.

### Asset and network sediment detention efficiency over multiple events

Asset-specific deposition and suspended sediment concentrations taken across the monitoring period provide SuDS asset fine sediment detention efficiencies. The asset overview presented in Figs. 2 and 4 illustrates that on this site, and within this SuDS network, the linear wetland is the most efficient of the SuDS assets compared. The linear wetland, despite being located second in the SuDS network (third for car park-sourced sediment), is more efficient in temporary bed deposition of REO tagged sediment (time- and location-specific released material) and urban sediment pollutants in general. The linear wetland also has a higher TSS treatment efficiency, but with acknowledgement that there is a greater TSS mitigation range (both beneficial and detrimental).

The tagged sediment transport dataset has been disaggregated by SuDS asset, allowing the detention efficiency of each asset to be calculated over the 52 weeks of monitoring. The detention efficiencies are presented in Table 2, which illustrates that the linear wetland and swales are the more effective assets in temporary, multiple rainfall–runoff event fine sediment detention.

**Table 2:** SuDS asset sediment detention efficiency (%)

Monitoring period	Release	Week 2	Week 8	Week 16	Week 24	Week 32	Week 40	Week 48	Week 52
Asset									
Wetland	0	94	90	78	73	66	37	49	46
Linear wetland	0	82	75	78	72	77	70	75	70
Short swale	0	82	85	75	74	68	63	73	71
Long swale	0	88	82	87	85	84	79	71	69

The tabulated values are for the average tagged sediment removal efficiency (%) rather than total sediment removal

Both linear wetland and swale assets are illustrated to be ~25% more efficient than the wetland asset. This is surprising as the wetland, with a greater detention time, would be expected to support fine sediment settling. However, both the linear wetland and swale function to convey stormwater at or below the height of the assets’ vegetation. Both linear wetland and swale therefore have a higher Manning ‘n’ and unit width of vegetation blocking the flow path (Deletic 2004). The vegetation density and height (comparable to the stormwater surface water level) beneficially influence the sediment removal efficiency of these SuDS asset. Furthermore the wetland, a wet SuDS asset, is seen to be less effective in the longer term than the swales and linear wetland. This supports the hypothesis that sediment held in wet assets has a proportion of material in suspension and thus this sediment is more easily transported during rainfall–runoff events. Movement of sediment in suspension requires limited entrainment effort and therefore can potentially be transported faster or further than deposited sediment.

### Suspended and deposited sediment load movement driving factors

Correlation and regression analysis of the field data provides an insight into the linkages, influencing factors and relationships between the environmental conditions and changing suspended sediment concentration or deposition. Sediment settling velocity is driven by particle size and flow characteristics (velocity, turbulence, transport capacity) (Beuselinck et al. 1999), so field rainfall–runoff and flow characteristics were compared to tagged and total sediment concentrations.

Table 3 illustrates the distribution of each of the factors under consideration in suspended sediment concentration or deposition analysis. It is noted that not all datasets are Gaussian. All factors show a skew greater than 0 and a level of kurtosis. However, only datasets with a skew or kurtosis greater than two standard deviations were considered non-Gaussian (Tabachnick and Fidell 1996).

**Table 3:** Distribution analysis of sediment transport factors

Factors	Mean	Median	Skewness	Kurtosis
Percentage detention efficiency (%)	71.88	75.23	−1.10	0.64
Cumulative ADD (h)	**4655**	**2798**	**1.43** ^*^	**0.46**
ADD since last sample (h)	**21**	**8**	**1.3** ^*^	**1.6** ^*^
Wetted surface (m^2^)	**1.78**	**0.72**	**1.16** ^*^	**−0.56**
ω stream power per unit channel length (W/m)	**38.23**	**4.31**	**3.33** ^*^	**11.74** ^*^
Cumulative total rainfall depth (mm)	448	476	0.11	−1.19
Cumulative number of rainfall events (no.)	**1587**	**1338**	**0.42** ^*^	−**1.11**
Velocity max (m/s)	0.5	0.34	0.13	−1.73
Running average velocity (m/s)	0.28	0.08	0.07	−1.94
Average velocity (m/s)	**0.26**	**0.11**	**0.54** ^*^	**−0.90**
Average depth (m)	**0.28**	**0.29**	**0.50** ^*^	**0.02**
Re	**210**	**121**	**0.55** ^*^	**−1.52**
Fr	0.17	0.09	0.28	−1.57

Factors highlighted bold are non-Gaussian datasets, with the deviance from Gaussian distribution highlighted by asterisk

There is a strong correlation between wetland deposition and both cumulative ADD and rainfall depth of the event prior to sampling. These factors are also shown to correlate to the short-swale detention efficiency, alongside the maximum velocity occurring within the monitoring period. The linear wetland dataset appears most closely aligned with the maximum velocity occurring prior to the sampling period. All factors listed in Table 4 show a correlation >0.2 to either the total network or a specific SuDS asset, with the exception of ADD since the last sample.

**Table 4:** Correlation coefficients for sediment transport factors

Percentage detention efficiency	Total network	Wetland	Linear wetland	Long swale	Short swale
Pearson’s correlation coefficient					
Cumulative depth of rainfall	0.13	**0.31**	−0.29	−0.06	**0.36**
Velocity max	−0.23	**−0.37**	**−0.52**	−0.08	*−0.69*
Running average velocity	−0.24	0.10	−0.17	0.01	−0.05
Fr (Froude number)	**0.34**	**0.42**	−0.25	−0.06	**0.33**
Spearman’s Rho correlation					
Cumulative ADD	−0.20	*−0.95*	0.11	0.28	*−0.75*
ADD since last sample	−0.06	−0.12	−0.05	0.01	−0.05
Wetted surface area	**0.43**	**0.55**	−0.23	−0.15	**0.36**
Stream power	0.09	**0.55**	−0.23	−0.15	**0.36**
Last prior event depth of rainfall	−0.17	*−0.96*	**0.40**	**0.36**	*−0.75*
Average velocity	−0.30	0.05	−0.28	−0.20	**−0.52**
Average depth	0.28	0.15	−0.26	−0.20	**−0.52**
Re (Reynold’s number)	−0.12	**0.55**	−0.23	−0.15	**0.36**

Values highlighted in bold show moderate correlation (0.3–0.6), and values in italics show strong correlation (>0.6). All factors are as at the time of sampling unless otherwise stated

Extending the correlation findings, regression analysis of the field data defined a linear relationship between total suspended sediment concentration, flow velocity and depth. It is expected that the total bed load concentration be defined to some extent by the suspended sediment concentration (both tagged and total). Writing total bed deposition as a function of suspended sediment concentrations, the following empirical description can be derived from the field data (*p* < 0.01, *R* ^2^ > 0.8):

1 $$\text{Total bed deposition} = f(PR + P^{2} + R^{2} )^{2}$$

where the total bed deposition is the mass within the sediment trap at a specific monitoring location, *P* is the TSS at the monitoring point under analysis, and *R* is the REO suspended sediment concentration at the same location and sample period.

Taking into consideration all factors that have a correlation of greater than 0.2, multivariate regression analysis was used to create a statistical description of the network fine sediment detention efficiency. The regression function achieving statistical significance (*p* < 0.0001) and relevant predictive capabilities (*R* ^2^ > 0.50, adj. *R* ^2^ = 0.49) is presented as Eq. 1.

2 $$\text{Detention efficiency} = f(ABH + EFG + B^{2} K + DH^{2} + J^{3} )$$

where *A* = Fr, *B* = Re, *D* = average velocity, *E* = running average velocity, *F* = velocity max, *G* = number of rainfall event, *H* = depth of rainfall, *J* = stream power and *K* = wetted surface areaAll of the factors represented in Eq. (2), with exception to *DH* ^2^, are significant within the regression model (*p* ≤ 0.01, *DH* ^2^ *p* = 0.06). River sediment conveyance is often estimated using stream power (ω), and stream power alone does not show a predictive function within this SuDS network. The regression function suggests that both flow inertia (Fr) and turbulence (Re) are significant in determining multiple event fine sediment transport, in conjunction with stream power.

The SuDS network and asset detention efficiency has several non-normal distributed factors in the dataset (Table 3). Thus, the generalised lineal model (GLM) was used in the regression analysis to provide a descriptive and predictive statement of detention efficiency trend. GLM, and specifically logistic regression, provides a structural component, linkage function and a response distribution relative to the response point in the covariate space. The key benefit of logit functionality is the inclusion of the link function. This allows the non-normal distribution response to be connected and respond to the structural factors in the regression analysis. Using the logit function in this regression analysis has allowed the key structural drivers (factors) in the sediment transport dataset that influence multiple event SuDS asset detention efficiency to be identified.

3 $$\text{Logit}\left( Y \right) = \text{Ln}\,(Y - 0.001/100 - Y)$$

where $Y = ABH + EFG + B^{2} K + DH^{2} + J^{3}$; the detention efficiency (%)

From the above regression analysis, it can be seen that the quantity of tagged sediment material deposited in the SuDS treatment train is a function of multiple runoff and flow parameters. The GLM regression between tagged sediment deposition and rainfall/flow factors provides a starting point for further detailed investigation into the empirical description of long-term sediment transport through a SuDS network. Equation 1 illustrates that both rainfall–runoff event characteristics and the concentration of suspended sediment material influence the tagged sediment bed deposition. While the logistical function of tagged sediment detention efficiency model is significant (*p* < 0.0001), the predictive function is only moderate (0.5 < *R* ^2^ < 0.8). Therefore, there is greater complexity in the internal sediment transport of this tagged material than the above direct relationships can describe.

## Conclusion

Urban fine sediment pollution has been shown to move within and through a SuDS network as a result of multiple rainfall–runoff events. This field research has illustrated that urban fine sediment from a single sediment release continues to be transported through this SuDS network for 24–52 weeks after wash-off (limited by sampling period in this field work). The assumption that urban sediment pollution is captured and permanently retained by a SuDS asset during the initial event is therefore inaccurate, and an element of hysteresis occurs within SuDS sediment treatment and transport.

The TSS and sediment trapping (deposition) efficiencies of each SuDS asset within the J4M8 SuDS networks vary. The linear wetland has been shown to function effectively as a sediment trapping mechanism (higher sediment deposition rate and detection efficiency percentage) and TSS mitigation measure (change between upstream and downstream surface sample concentrations). Both linear wetland and swale fine sediment detention efficiencies illustrate the beneficial influence of ephemeral vegetated treatment measures, resulting in higher treatment efficiencies overall and more consistent sediment bed deposition.

The particle size distribution of detained (deposition) sediment decreases in primary and d50 particle size through the SuDS network. While this may result in part to the design of individual assets, it does support the theory of SuDS treatment train implementation, the use of multiple, connected SuDS assets, to achieve greater overall stormwater quality improvement. Thus, while these field data show that up to 17% of the released sediment was conveyed downstream of the SuDS network (respective of the source location and SuDS network composition), the inclusion of multiple SuDS assets resulted in a notably finer particle size detention that an individual SuDS asset alone.

Initial regression analysis of the field data suggests the multiple rainfall–runoff transport processes of urban sediment pollution through SuDS networks are complex. While the expected relationships between rainfall–runoff and flow characteristics describe the TSS and total bed deposition occurring fortnightly within this SuDS network, the movement of deposited tagged sediment is less easily defined. Equation 2 provides an insight into the key drivers of fine sediment resuspension and deposition within this SuDS network, connecting the single release tagged sediment movement to flow and runoff event characteristics. However, further modelling of the field results is required to describe in detail the fine sediment transport processes of individual sediment release fate within a SuDS network, and therefore the longer-term influence of sediment and flow hysteresis on SuDS water quality improvement efficiencies.
